# Complexity of the 5′UTR region of the *CLCN5* gene: eleven 5′UTR ends are differentially expressed in the human kidney

**DOI:** 10.1186/1755-8794-7-41

**Published:** 2014-07-07

**Authors:** Enrica Tosetto, Alberto Casarin, Leonardo Salviati, Alessandra Familiari, John C Lieske, Franca Anglani

**Affiliations:** 1 Laboratory of Histomorphology and Molecular Biology of the Kidney, Department of Medicine DIMED, University of Padova, via Giustiniani, 2, 35128 Padova, (PD), Italy; 2 Clinical Genetics Unit, Department of Pediatrics, University of Padova, Padova, Italy; 3 Department of Internal Medicine, Division of Nephrology and Hypertension, and Department of Laboratory Medicine and Pathology, Mayo Clinic, Rochester, MN, USA

**Keywords:** Dent disease, *CLCN5* gene, 5*’*UTR isoforms, mRNA variant, Gene expression

## Abstract

**Background:**

Dent disease 1 represents a hereditary disorder of renal tubular epithelial function associated with mutations in the *CLCN5* gene that encoded the ClC-5 Cl^-^/H^+^ antiporter. All of the reported disease-causing mutations are localized in the coding region except for one recently identified in the 5*’*UTR region of a single patient. This finding highlighted the possible role for genetic variability in this region in the pathogenesis of Dent disease 1.

The structural complexity of the *CLCN5* 5*’*UTR region has not yet been fully characterized. To date 6 different 5*’* alternatively used exons - 1a, 1b, 1b1 and I-IV with an alternatively spliced exon II (IIa, IIb) - have been described, but their significance and differential expression in the human kidney have not been investigated. Therefore our aim was to better characterize the *CLCN5* 5*’*UTR region in the human kidney and other tissues.

**Methods:**

To clone more of the 5*’* end portion of the human *CLCN5* cDNA, total human kidney RNA was utilized as template and RNA ligase-mediated rapid amplification of cDNA 5*’* ends was applied.

The expression of the different *CLCN5* isoforms was studied in the kidney, leucocytes and in different tissues by quantitative comparative RT/PCR and Real -Time RT/PCR.

**Results:**

Eleven transcripts initiating at 3 different nucleotide positions having 3 distinct promoters of varying strength were identified. Previously identified 5*’*UTR isoforms were confirmed, but their ends were extended. Six additional 5*’*UTR ends characterized by the presence of new untranslated exons (c, V and VI) were also identified. Exon c originates exon c.1 by alternative splicing. The kidney uniquely expresses all isoforms, and the isoform containing exon c appears kidney specific. The most abundant isoforms contain exon 1a, exon IIa and exons 1b1 and c. ORF analysis predicts that all isoforms except 3 encode for the canonical 746 amino acid ClC-5 protein.

**Conclusions:**

Our results confirm the structural complexity of the *CLCN5* 5*’*UTR region. Characterization of this crucial region could allow a clear genetic classification of a greater number of Dent disease patients, but also provide the basis for highlighting some as yet unexplored functions of the ClC-5 proton exchanger.

## Background

Dent disease 1 (OMIM 300009) is an X-linked recessive disorder of renal tubular epithelial function, associated with genetic variation of the *CLCN5* gene that encodes the ClC-5 Cl^-^/H^+^ antiporter. The disease is characterized by low-molecular-weight proteinuria, hypercalciuria, nephrocalcinosis, nephrolithiasis and one or several features of proximal tubular dysfunction (glycosuria, aminoaciduria, and phosphaturia, etc.) [1]. ClC-5 is expressed in the kidney, particularly in proximal tubular cells and intercalated collecting duct cells. The human *CLCN5* gene, spanning about 170 kb of genomic DNA on chromosome Xp11.23/p11.22, consists of 17 exons including 11 coding exons (2-12) and 6 different 5′ alternatively used exons (5′UTR), some remaining untranslated [1-4]. Transcripts including the untranslated exon 1a (NM_000084.4: mRNA variant 3) [2] or 1b (NM_001282163.1: mRNA variant 4) [3] are spliced to exon 2 and contain the start sequence ATG. A third mRNA comprises a larger exon 1b and retains intron 1 (exon 1b1) (alternative mRNA variant 4) [4]. Subsequently, two additional long transcripts due to alternative splicing of exon II and including exons I to IV have also been identified (NM_001127899.3: mRNA variant 1 and NM_001127898.3: mRNA variant 2) [5]. Both of these transcripts carry the ATG start sequence in exon III, thereby encoding a NH_2_-terminal extended ClC-5 isoform consisting of 816 amino acids instead of the canonical transcript of 746 amino acids.

Although more than 150 different mutations have been reported within the *CLCN5* coding exons, patients with typical symptoms of Dent disease 1 have been genotyped by our group and others in whom no mutations could be detected [6-9]. The presence of many different 5′UTR ends of *CLCN5* mRNA in the kidney highlights the complexity of both the molecular structure and the regulatory apparatus of the gene. Moreover, the 5′UTR might hide Dent disease 1 disease-causing mutations or polymorphisms that may influence disease expression. Indeed, while analysing 30 *CLCN5* negative patients our group identified a nucleotide substitution in the 5′ untranslated exon 1b1 of one individual which appeared disease-causing since it was not detected in 471 X normal chromosomes [10].

The functional significance of these regulatory regions has not been elucidated. Their differential expression in the kidney versus other human tissues like brain, skeletal muscle and the eye has also not been assessed, despite the potential involvement of these organs in Dent 1 cases [11]. Thus we decided to study this region in depth. Here we report the identification of additional 5′UTR ends of human *CLCN5* cDNA within the kidney, including the presence of newly identified exons. In total eight exons are now known to be present in the 5′UTR region of the *CLCN5* gene, giving rise to eleven isoforms. Moreover we succeeded in extending the 5′ ends of the previously known *CLCN5* transcripts to identify new transcription start sites. These novel *CLCN5* mRNA species are demonstrated to be differently expressed in kidney and other human tissues.

## Methods

### RNA ligase-mediated rapid amplification of cDNA 5′ ends PCR

The GeneRacer kit (Invitrogen) was used in accordance with the manufacturer’s instructions to obtain clones with the 5′ portion of the human *CLCN5* cDNA. In brief, 5 μg of total human kidney RNA (Stratagene) was treated with calf intestinal phosphatase to remove 5′ phosphates. This has no effect on capped full-length mRNA but removes non-mRNA or truncated mRNA from the ligation reaction. The sample was then treated with tobacco acid pyrophosphatase to remove the 5′ mRNA cap structure, which leaves a 5′ phosphate required for ligation to the GeneRacer RNA Oligo (5′-CGACUGGAGCACGAGGACACUGACAUGGACUGAAGGAGUAGAAA-3′). The GeneRacer RNA Oligo was ligated to the 5′ end of the decapped mRNA with T4 RNA ligase, which provides a known priming site for the GeneRacer PCR primers. A reverse transcription reaction was then performed using SuperScript III Reverse Transcriptase and the GeneRacer Oligo dT Primer (5′-GCTGTCAACGATACGCTACGTAACGGCATGACAGTG(T)24-3′) provided in the kit. The *CLCN5* 5′ cDNA ends were amplified with a touchdown PCR using a *CLCN5* gene specific antisense primer (rRACE Ex 2), the GeneRacer 5′ primer and Platinum Taq DNA Polymerase High Fidelity (Invitrogen). Cycling conditions followed the manufacturer’s protocol. After the successful amplification was confirmed by agarose gel electrophoresis, a second round of nested PCR amplification was done as above, except that the GeneRacer 5′ nested primer and the *CLCN5* gene specific nested antisense primers were used in place of the GeneRacer 5′ primer and the *CLCN5* rRACE Ex 2 primer, respectively (see Additional file 1). The PCR products were cloned into plasmid vector pCR4-TOPO and transformed into competent One Shot TOP10 cells with a TOPO TA cloning kit (Invitrogen) following the supplier’s protocol. Colonies were analysed by PCR, using vector specific primers (M13F: 5′-GTAAAACGACGGCCAG-3′; M13R: 5′-CAGGAAACAGCTATGAC-3′). In total, 172 clones were sequenced using vector specific primers.

### Sequencing

The sequence analysis of cDNA clones and of RT/PCR products was performed using a direct Sanger sequencing method. The sequencing process included purification of PCR products using the MinElute PCR Purification Kit (Qiagen), sequencing via the Big Dye Terminators v1.1 Cycle Sequencing Kit (Applied BioSystems), and final purification using Centrisep Columns (Princeton Separation), all in accordance with operational manuals. Sequences were analyzed using an ABI-PRISM 3100 Genetic Analyzer (Applied BioSystems). To compare *CLCN5* 5′UTR cDNAs and coding region with its genomic sequences, the NCBI Blast 2 sequence alignment program was used. The human *CLCN5* mRNA (GeneBank accession number NM_001127899.3; NM_001127898.3; NM_000084.4; NM_001282163.1 and NM_001272102.1), the human *CLCN5* DNA sequence on chromosome X (GenBank accession number NG_007159.3) and the *CLCN5* promoter region (GeneBank accession number AB020597.1) were used for comparison.

### Quantitative comparative RT/PCR analysis of *CLCN5* mRNA in different human tissues

All patients gave their informed, written consent. Total RNA from leucocytes of healthy human subjects was extracted using the PAXgene blood RNA kit (Qiagen) according to the manufacturer’s instructions. The collection of the blood samples used in this study was made with the appropriate approval of the Ethics Committee of Azienda Ospedaliera of Padova. One μl of RNA was used for spectrophotometric quantification at 260 and 280 nm using the NanoDrop ND-1000 spectrophotometer (NanoDrop Technologies), and RNA integrity was checked with the Agilent 2100 bioanalyzer (Agilent Technologies). Total RNA from human normal tissue samples (kidney, brain, lung, liver, colon, placenta, testes, skeletal muscle), and from endothelial cells were purchased from Stratagene.

To analyze the differential expression of *CLCN5* gene 5′UTR ends, a different set of primers were designed (see Additional file 2). The primers Ex I/II F and Ex IV/2 R were the same used by Ludwig et al. [5]. Two hundred nanograms of total RNA were reverse-transcribed in a total volume of 20 μl containing 5 mM MgCl_2_, 1 mM dNTPs (Roche Diagnostics), 2.5 μM random hexamers (Applied Biosystems), 1U RNase inhibitor (Applied Biosystems), 2.5 MuLV reverse transcriptase (Applied Biosystems) in a buffer of 50 mM KCl, 10 mM Tris HCl pH 8.3. The reaction was carried out at 42°C for 30 min followed by 5 min at 99°C. An aliquot (1.5 μl) of RT reaction was used to amplify all alternative *CLCN5* mRNA species in a final volume of 25 μl containing 1.5, 2 or 3 mM MgCl_2_, 0.2 mM dNTPs (Roche Diagnostics), 0.4 μM primers, 0.04 U JumpStart Taq (Sigma-Aldrich) in 50 mM KCl, 10 mM Tris HCl pH 8.3. The amplification profile for each primer set consisted of an initial denaturation at 95°C for 5 min, followed by different amplification cycles (45 s at 94°C, 45 s at specific Ta°C, 1 min at 72°C), and an extension at 72°C for 7 min. PCR conditions are given in (see Additional file 2).

To quantify the relative expression of each 5′UTR end, a semiquantitative comparative RT/PCR approach was performed using *GAPDH* as a housekeeping gene [12]. RT/PCR products were analysed by 7% polyacrylamide gel electrophoresis followed by silver staining as previously described [12]. One μl was also analysed and quantified using the Agilent Bioanalyser technology (Agilent Technologies). Negative control reactions were performed without reverse transcriptase during a cDNA synthesis step to rule out any genomic contamination.

### Real-Time PCR of *CLCN5* mRNA in the human kidney

Real-Time quantitative polymerase chain reaction was performed to analyze the expression levels of all the *CLCN5* mRNA species in the human kidney. Changes in *CLCN5* gene mRNA levels were determined by quantitative relative Real-Time PCR with the iCycler Termal Cycler (Bio-Rad). The reaction was carried out in a final volume of 25 μl containing 1 μl of RT reaction, 1× iQ SYBR Green supermix (BioRad) and 0.3 μM of specific primers for 5′UTR exons. To avoid genomic contamination, primers spanning exon boundaries were constructed. It was not possible to design primer pairs specific for all isoforms, and for this reason isoforms containing exon IIa were quantified together as a group, as were isoforms containing exon IIb (see Additional file 3). Samples were loaded in triplicate, 25 μl/well, in 96-well plates (Biorad). Negative control reactions were performed to rule out any contamination.

The thermal cycling profile was the same for each primer set and consisted of an initial denaturation at 95°C for 5 min, followed by 45 amplification cycles of 10 s at 95°C and 45 s at 66°C, followed by 1 cycle at 60°C for 1 min. Melting curve analysis consisted of 80 cycles at 60°C for 10 s and was used to confirm the specificity of the amplification products. *GAPDH* was the housekeeping gene used as an internal primer control. A positive cDNA control was also used as reference (the isoform A, the most abundantly expressed).

The relative expression of each 5′ UTR isoform was compared to *GAPDH* expression calculating the ΔCt value of expression as follows: Ct isoform – Ct *GAPDH*. The Ct gives a raw idea about the fold change in gene expression [13].

### Bioinformatic analyses

The NNSPLICE (http://www.fruitfly.org/seq_tools/splice.html) [14], NetGene2 (http://www.cbs.dtu.dk/services/NetGene2/) [15], and GeneSplicer (http://www.cbcb.umd.edu/software/GeneSplicer/gene_spl.shtml) [16] programs were used to identify potential splice sites.

Amino acid sequences were subjected to computational analysis with the ORF Finder program (http://www.ncbi.nlm.nih.gov/projects/gorf/).

The online tool Human Splicing Finder version 2.4.1 (http://www.umd.be/HSF/HSF.html) was used to identify splicing motifs in our human sequence of interest [17]. The web server mRNAfold (http://rna.tbi.univie.ac.at/cgi-bin/RNAfold.cgi) was used to predict the pre-mRNA secondary structure [18,19].

The analysis of the *CLCN5* promoter region was performed using the ENCODE (Encyclopedia of DNA Elements) project [20] data in the UCSC Genome Browser for defining the transcriptional regulatory regions.

## Results

### Structure of *CLCN5* 5′UTR region

RACE analysis of *CLCN5* 5′ cDNA ends in the human kidney detected eleven transcripts initiating at three different nucleotides: - 2407, - 1426 in respect to the ATG initiation codon in exon 2, and at - 660 in respect to the ATG initiation codon in exon III. Analysis not only confirmed the presence of previously identified UTR isoforms (mRNA variants 1-4), but also extended their ends and identified new transcription start sites. A transcript initiating at nucleotide - 2407 corresponds to mRNA variant 3, but is extended a further 41 bp in respect to the +1 origin reported in the NCBI reference sequence NM_000084.4. This result is in agreement with *in silico* analysis conducted with NNSPLICE, NetGene2, or GENESPLICER [14-16]. All of the tested programs predict a consensus sequence (5′-tcccag^GC-3′) containing the conserved motif “AG” at 41 bp upstream of exon 1a, which constitutes a potential acceptor splice site of high strength. These same programs do not identify the previously described acceptor site.

**Figure 1:**
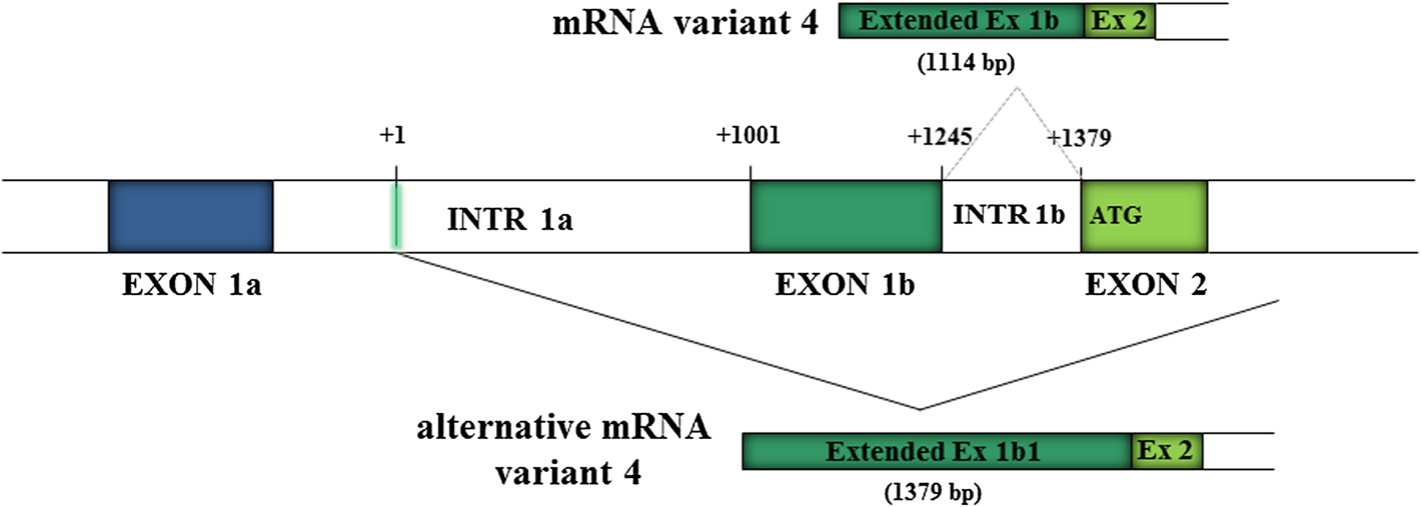
**Characterization of the *CLCN5 *5′UTR variant 4 and alternative variant 4 by RACE PCR and sequencing.** + 1 indicates the putative transcriptional start site of alternative variant 4, located at intron 1a, 1001 nt upstream of the first nucleotide of exon 1b described by Fisher et al. (2). For variant 4 it was not been possible to obtain the full length cDNA but probably the two variants have the same transcription start site and differ only regarding the absence/presence of intron 1b. Coloured boxes represent exons and open boxes represent introns.

We were also able to confirm the presence of the alternative mRNA variant 4, albeit longer than earlier described by Forino et al. [4]. It retains intron 1b and consists of an extended exon 1b (exon 1b1) with a new putative transcription start site located at nucleotide -1426 in intron 1a, 1001 nt upstream of the beginning of exon 1b, and is 1379 bp long. *In silico* analysis also detected a candidate acceptor splice site that contains the conserved motif “AG”, (5′-ctacag^AT-3′) corresponding to the beginning of the transcript identified by RACE analysis (Figure 1).We also extended the 5′ end of mRNA variant 4, 870 nt further in respect to the +1 origin reported in the NCBI reference sequence NM_001282163.1, but it was not possible to obtain the full length cDNA. Therefore we hypothesized that the mRNA variant 4 and the alternative mRNA variant 4 have the same transcription start site differing only regarding the absence/presence of intron 1b (Figure 1).

We confirmed the presence of the mRNA variants 1 and 2 identified by Ludwig et al. [5] with both alternatively spliced exons IIa and IIb which, according to the NCBI reference sequences NM_001127899.3 and NM_001127898.3, contain the transcriptional start site at nucleotide - 660 in respect to the ATG initiation codon in exon III.

**Figure 2:**
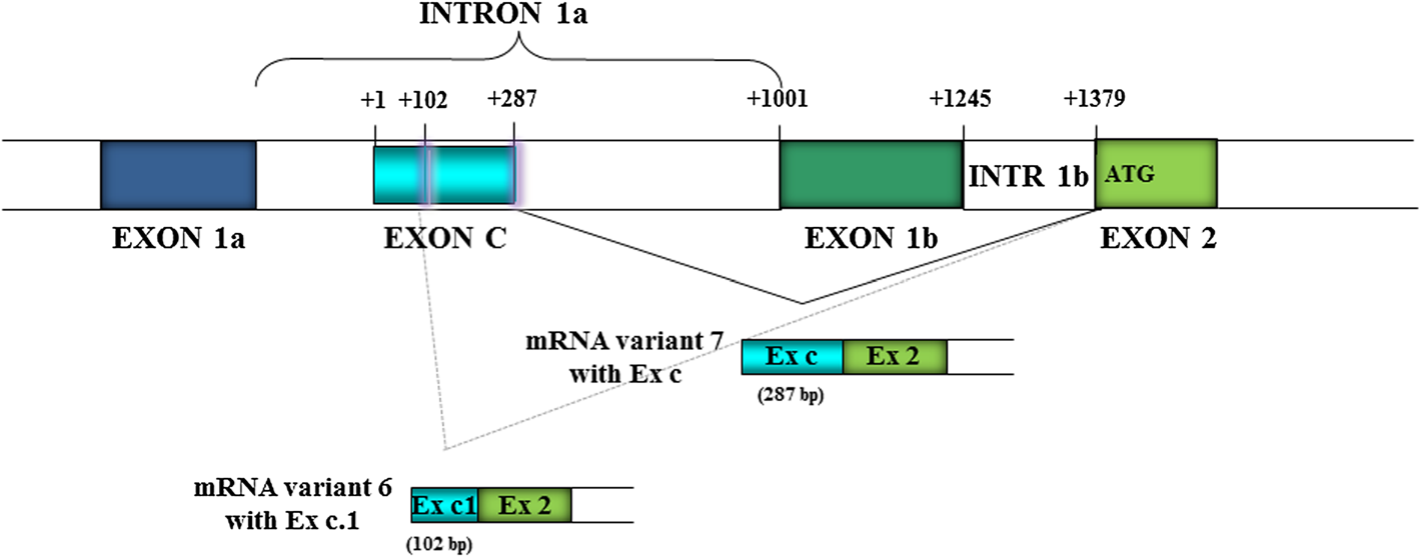
**Characterization of the *CLCN5 *5′UTR variants 6 and 7 by RACE PCR and sequencing.** + 1, which is located at intron 1a, indicates the putative mRNA variant 7 transcriptional start site that is the same of alternative variant 4. Exon c of 287 bp after 5*’* alternative splicing originates two different mRNAs containing an exon c.1 of 102 bp (variant 6) and exon c of 287 bp (variant 7). Coloured boxes represent exons and open boxes represent introns.

**Figure 3:**
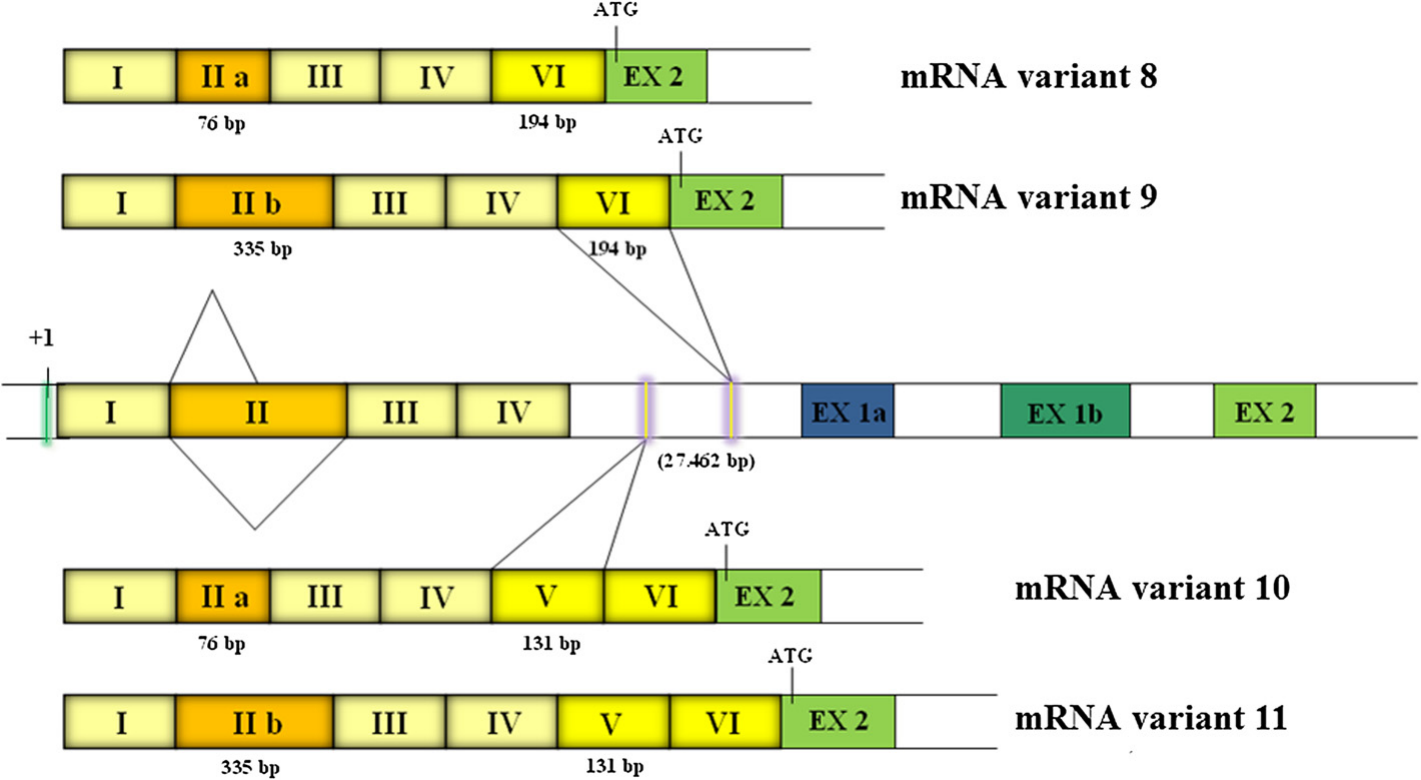
**Characterization of the new *CLCN5 *5′UTR long transcripts by RACE PCR and sequencing.** The four long transcripts contain the already described exons I-IV and the new exons V and VI (variants 8-11) (131 bp and 194 bp long, respectively), located 7820 bp and 11977 bp downstream of the exon IV respectively . + 1 indicates the putative transcriptional start site located at nucleotide - 660 in respect to the ATG initiation codon in exon III. Coloured boxes represent exons and open boxes represent introns.

RACE analysis detected six new 5′UTR ends of human *CLCN5* cDNA. One isoform consisted of a new 287 bp exon newly named exon c, located within intron 1a according to the classical 5′UTR structure, representing an alternative splicing of the mRNA alternative variant 4 if one considers the new structure of exon 1b1 resulting from our RACE PCR analysis. Exon c contains a palindromic region of 28 nucleotides and, as a result of 5′ alternative splicing, originates two different mRNAs containing an exon c.1 of 102 bp (5′-TT^gtaagt-3′) and exon c (5′-AG^gttggt-3′), respectively (Figure 2). We called these variants 6 and 7. As observed from analysis of splice sites and from consensus values calculated with the Shapiro and Senapathy matrices [21], both donor splice sites have similar consensus values (c.1 79.4, and c 79.2), and have similar strength, and likely compete for splicing factors.Due to alternative splicing of exon II, four new long transcripts were detected, two of them containing the exon VI (variant 8 and 9), and the other two containing exon V and VI (variant 10 and 11). Both new exons V and VI (131 bp and 194 bp long, respectively) are located 7820 bp and 11977 bp downstream of the exon IV respectively (Figure 3). Bioinformatic tools identified the acceptor (5′-tgtcag^AG-3′) and donor (5′-AG^gtaagc-3′) splice sites upstream and downstream of the exon VI. These tools identified only the donor splice site (5′-AG^gtatgt-3′) for exon V.

**Figure 4:**
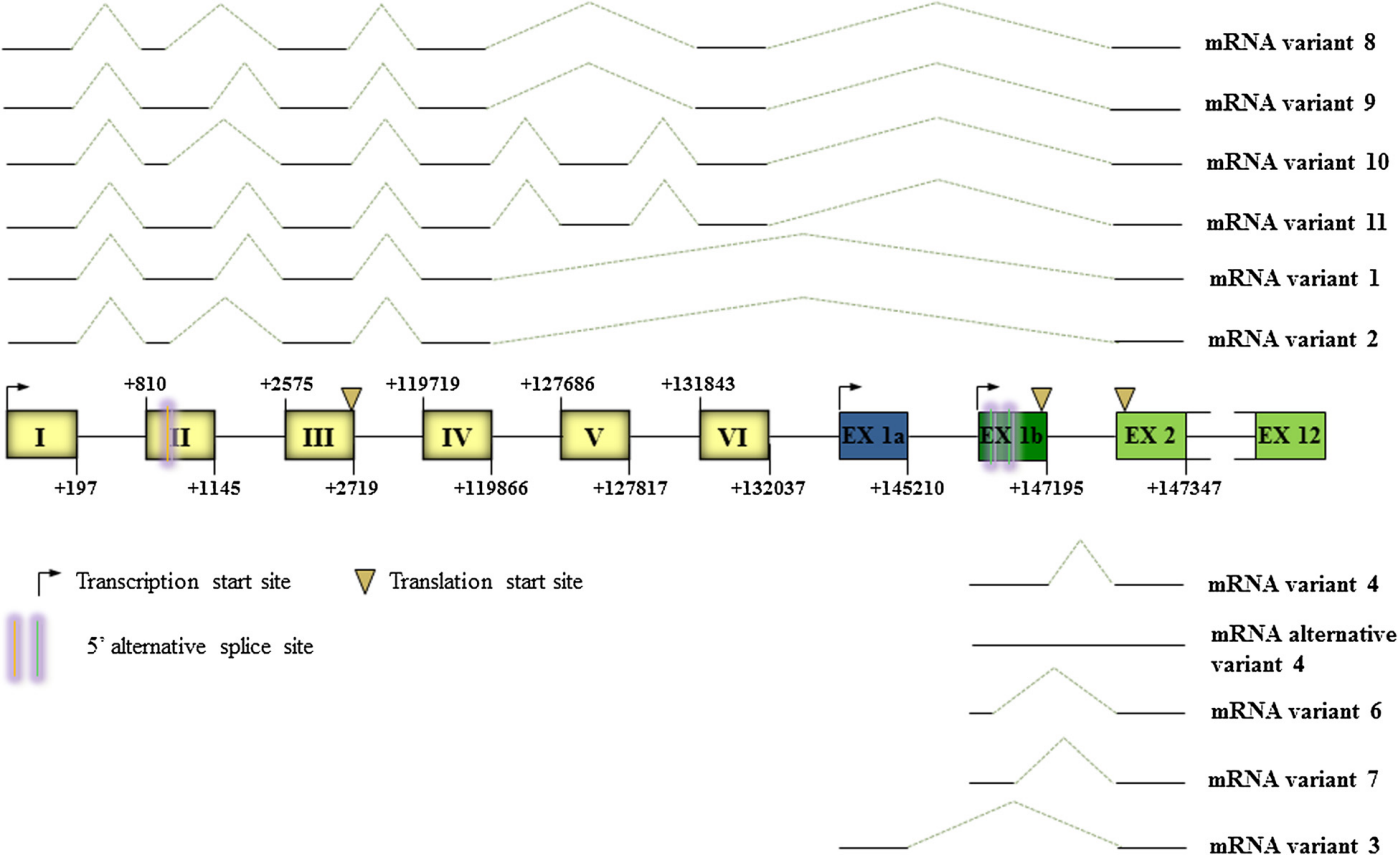
**Genomic organization of *CLCN5 *5′UTR region.** The *CLCN5* 5*’* UTR region consists of 8 different 5*’* alternatively used exons, some of this, remains untranslated. As a result of 5*’* alternative splicing (represented by two vertical bars) in exons II and 1b, 11 different mRNAs are generated. Transcription initiates from three different start sites (represented by an arrow) and there are three translation start sites (represented by an inverted triangle). Coloured boxes represent exons and the connecting lines between boxes the introns.

With our experiments we were not able to identify the mRNA variant 5 (NM_001272102.1). From these results we can conclude that the human *CLCN5* gene comprises at least 20 exons, eight of them in the 5′UTR region, with transcription initiating from at least three different start sites. As a result of 5′ alternative splicing in some exons, 11 different mRNAs are generated (Figure 4). The complete sequence of extended 5′ UTR ends of the known *CLCN5* transcripts (type 3, 4 and alternative 4) and of the newly identified *CLCN5* 5′UTR variants (types 6-11) are given in Additional file 4.

### Expression of *CLCN5* 5′UTR ends in different human tissues

Healthy human tissues (kidney, brain, lung, liver, colon, placenta, testes, skeletal muscle, endothelial cells and peripheral leucocytes) from normal individuals were used to evaluate the presence and the expression of the different *CLCN5* transcripts. Both the translated region common to all isoforms (exons 3-6) and the 5′UTR ends were analyzed. Quantitative comparative RT/PCR analysis detected *CLCN5* mRNA in all investigated tissues (Table 1, and Additional file 5).

**Table 1:** **Results of RT/PCR analysis of 5′ *CLCN5 *** **mRNA variants**

**Tissue**	**mRNA variants**											
	**1**	**2**	**3**	**4**	**Alternative 4**	**6**	**7**	**8**	**9**	**10**	**11**	***CLCN5***
												**Common**
												**Region**
**Liver**	+++	+/-	+++	-	++	+	-	-	-	-	**-**	+++
**Lung**	+++	+/-	+	-	++	+	-		-	-	**-**	++
**Placenta**	+++	+	++	-	+	-	-	+++	-	-	**-**	+
**Testes**	+++	+	+++	-	+++	-	-	+++	-	+++	**-**	+++
**Skeletal muscle**	+++	+	++	-	++	-	-	+++	-	-	-	+++
**Brain**	++	+	+++	-	+++	-	-	+++	-	-	-	++
**Colon**	+++	+	+++	+	+++	-	-	++	++	+	+	+++
**Endothelial cells**	+++	+/-	++	-	+	-	-	-	-	-	-	+
**Kidney**	+++	+	+++	-	+++	++	+++	+++	++	++	+	+++
**Leucocytes**	++	+	++	-	++	-	-	+++	-	-	-	++

The table reports the presence/absence of all identified variants and their relative expression in different human tissues and cells.

Using primers specific for each 5′UTR isoform we demonstrated that some are not present in all tissues, and that the kidney is the unique tissue in which all isoforms are expressed (Table 1). Analysis of the distribution of *CLCN5* 5′UTR ends revealed that the already known mRNA variants 1-4 and alternative variant 4 are expressed in all tissues with variable levels: the mRNA variant 3 is the most abundant in all tissues except the lung, and the mRNA variant 4 is the less abundant in all tissues except the colon. mRNA variants 1 and 2 are also present in all tissues, and the transcript containing exon IIa is much more abundant than that with exon IIb.

The newly identified variants 6 and 7 are the most heterogeneous between different tissue samples: both are expressed in the kidney, only variant 6 was expressed in liver and lung, while neither variant 7 was present in the remaining tissues. The variant 7 appeared specific to the kidney and was absent from all other tissues. The four new long 5′UTR isoforms containing exon VI or exon V plus exon VI also have variable expression levels between tissues. The transcript with exon VI (variants 8 and 9) is present in almost all sites except liver, lung and endothelial cells, whereas the transcript with exons V and VI (variants 10 and 11) is expressed only in kidney, colon and in testes. In both cases the mRNAs with exon IIa are much more abundant than that with exon IIb (Table 1).

In summary, the different 5′UTR ends have variable expression levels from tissue to tissue. The tissues that are most similar regarding the isoform expression, both in terms of abundance and expression pattern, are kidney, colon and testis. The mRNA variants 3,2, the alternative variant 4 and variant 7 appear to be the most abundant in these 3 sites.

### Real-Time PCR quantification of the *CLCN5* mRNA species in the human kidney

**Figure 5:**
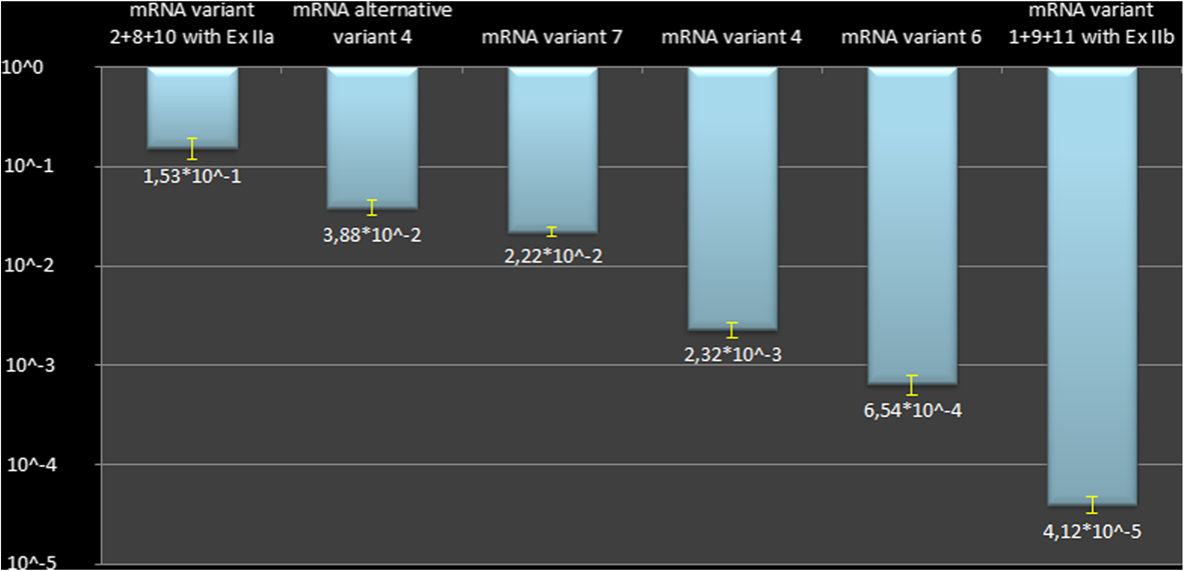
**Real time PCR quantification of *CLCN5 *isoforms in the human kidney.** Data were normalized to *GAPDH* housekeeping gene; the isoforms were quantified using the mRNA variant 3, the most abundantly expressed, as a reference value (10^0). The y- axis reports the expression values in logarithmic scale.

Finally, the levels of all 5′UTR isoforms we identified were quantified in human kidney tissue using Real Time PCR. The isoforms have very different expression levels: the mRNA variant 3, which was used as a calibrator to calculate the relative abundance of other isoforms, is the most abundant; all the mRNA variants containing exon IIa (variant 2, 8 and 10) and variant 7 have slightly lower levels (Figure 5).

In general, the results of Real-Time experiments were in agreement with those observed by RACE analysis. In fact out of 172 cloned 5′ RACE fragments, 77 (45%) contained type 3 mRNA species, 46 (27%) contained type 7, 34 (20%) were type 2, and 6 (3.5%) were alternative type 4.

## Discussion

Our results show that the human *CLCN5* gene comprises at least 20 exons, eight of them being in the 5′UTR region, with transcription initiating from at least three different start sites. As a result of 5′ alternative splicing in some exons, 11 different mRNAs are generated. Our findings highlight the structural complexity of the *CLCN5* 5′UTR region in renal and extrarenal tissues, and suggest that this region is likely involved in ClC-5 expression and Dent disease pathogenesis. Although some authors [5,8] have tested the promoter region in their patients without detecting variants, the deeper characterization obtained by our study should allow to explore regions of the gene never analyzed before for searching possible rare variants that may act as disease-causing mutations or modifier alleles.

To further characterize the functional organization of the gene, the 5′-flanking region of exons 1a, 1b1 and I were analyzed for possible promoter regions and transcription factor binding sites. ENCODE project data, aimed to delineate all of the functional elements encoded in the human genome sequence including the mapping of histone modifications, the transcription factor (TF) binding sites by chromatin immunoprecipitation (ChIP), and the transcriptional regulatory regions, were used. Thus the *Transcription* track, the *Overlayed H3K4Me1* and *Overlayed H3K27Ac* tracks, the *DNase Clusters*, and the *Txn Factor ChIP* tracks were considered. These tracks complement each other and together can shed much light on regulatory DNA [20].

The results suggest that 3 functional promoters are present in the *CLCN5* gene of different strength, originating all isoforms with varying efficiency. Strong promoters are present upstream of exon 1a and exon I, and indeed variant 3 and variants 1 and 2 are the mRNA species most expressed in the kidney (Figure 5). Both promoters lack characteristic features of eukaryotic promoters, but instead contain consensus binding sites for transcription factors. GATA1 and GATA2 factor binding sites and consensus binding sites for different transcription factors including E2F1 (transcription factor 1), ZNF263 (zinc finger protein 263), Nrf1 (nuclear respiratory factor 1), HMGN3 (high mobility group nucleosomal binding domain 3), USF1 (upstream transcription factor 1) and Ini1 (RING finger-like protein Ini1) are present for mRNA variants 1 and 2. For mRNA variant 3 the identified binding sites for the transcription factors are USF1, USF2 (upstream transcription factor 2, c-fos interacting), KAP1 (kinesin-ii-associated protein), and CTCF (CCCTC-binding factor). All these transcription factors were identified in a kidney cell line (HEK293). A weaker promoter appears to control the expression of mRNA variant 4, alternative variant 4 and variants 6 and 7. This promoter contains consensus binding sites for some transcription factors such as FOXA2 (forkhead box A2) and SETDB1 (SET domain, bifurcated 1). For all promoters the specific region containing the transcription factor binding sites overlap with a region that is DNaseI sensitive. At the functional level, DNase hypersensitivity suggests that a region is very likely to be regulatory in nature, and promoters are particularly DNase sensitive.

The data from ENCODE project did not identify sites for the transcription factor HNF1α. Instead, *in silico* analysis conducted by Tanaka et al. [22] had revealed numerous HNF1α binding sites in the 5′ regulatory sequences of both mouse and human *Clcn5/CLCN5* gene. The transactivation of the *Clcn5/CLCN5* promoter by HNF1α was verified in vitro, and the binding of HNF1α to the *Clcn5* promoter in vivo was confirmed by chromatin immunoprecipitation in mouse kidney [22].

The mRNA variant 4, the alternative variant 4 and variants 6 and 7 share the same transcription start site but have different lengths that depend on which donor site is used. They are probably generated, with differential efficiency, by multiple alternative splicing occurring at the 5′of a single exon. This type of exon commonly originates from ancestral constitutive exons that, following mutation/s inside the exon or along the flanking intron, result in the creation of new alternative splice sites that compete with the ancient one for splice site selection [23].

In the case of variants 6 and 7, the two alternative 5′ splicing sites in exon c have similar strength and so regulation is essential. It seems that the delicate balance between *cis* acting elements-enhancer (ESE) splicing regulatory elements (ESR) and silencers (ESS) located immediately upstream of each splice site is probably the major factor governing the level of each site usage in splicing [23,24]. In order to determine if this is the case, bioinformatic analysis, using the Human Splicing Finder version 2.4.1 program [17] was performed. The results of this analysis demonstrated that two ESE, two ESR and six ESS are present in the first 15 nt upstream of the donor splice site of exon c.1. Upstream of the donor splice site of exon c eight ESE, three ESR and five ESS are present. Therefore, although both sites have a similar strength, a higher density of ESE-ESR and lower density of ESS upstream of the exon c promote use of this splice site. Consistent with this observation, the expression levels of variant 7 are higher than those of variant 6 (Figure 5).In the case of mRNA variant 4, the donor splice site of exon 1b has a higher strength (value of 82.4) and therefore is favored. This isoform also contains 10ESE, 2 ESR and 6 ESS. However this is not in agreement with our experimental results because this isoform is barely expressed. Alternative variant 4, whose level of expression follows that of mRNA variant 3 of mRNA variants 1, 2 and 8-11, is characterized by the presence of exon 1b and the retention of intron 1b (exon 1b1) that, contrary to what usually happens, has not been removed during the processing of the primary transcript to mature messenger. Most likely other factors play an important role in the regulation of its transcription levels. Exon 1b1 could represent the ancestral constitutive exon from which all other exons (1b, c and c.1) originated (Figure 4). This exon is, in fact, usually present as the main product in respect to the others (Figure 5).

The GC content around splice sites is closely associated with the splice site usage [18,19,25]. We considered a region of 141 nucleotides surrounding the donors splice sites of exons c.1, c and 1b (70 nucleotides upstream and downstream of the splice site). It was possible to see that the highest GC content (11 GC) is in the donor splice site of exon c, exon c.1 (7 GC), and exon 1b (5 GC).

The web server mRNAfold was then used to predict the pre-mRNA secondary structure via calculation of minimum free energy [18,19]. It has been reported that local RNA secondary structures affect splice site selection, the splicing sites closest to the start transcription site forming more stable structures than those located in more central RNA locations [18,19]. The minimum free energy calculated by the software was -44.70, -38.84 and -30.70 kcal/mol for the donor splice sites downstream of the exons c, c1 and 1b, respectively. Both the evaluation of GC content and the calculation of free energy once again are in agreement with the results we obtained from the expression study. In fact, the expression level of isoform containing exon c is higher than those containing exon c.1 and 1b (Figure 5).

To conclude our characterization, we proceeded with the open reading frame analysis using the ORF Finder program. ORF analysis revealed that the mRNA variants 3, 6, 7 and alternative variant 4, as well as variants 8-11 encode for the canonical ClC-5 protein of 746 amino acids while variant 4, and variants 1-2 for a protein with 20 and 70 additional in frame amino acids, respectively. It is of note that the presence in the long transcripts of exon VI and/or V stabilizes the initiation of translation to the ATG in exon 2 and do not add, to the protein, additional amino acids. This is the most common situation among most genes that have alternative promoters and, while not generating different protein isoforms, have mRNA variants which differ in the transcription pattern and in translation efficiency.

The ClC-5 translated region was expressed in all human tissues examined. Our results are in agreement with what reported by Steinmeyer et al. [26] who demonstrated in the mouse that ClC-5 was predominantly expressed in the kidney but also observed in brain, liver, lung, and testis. Unlike Ludwig et al. [5], but in agreement with Ramos-Trujillo et al. [27] we demonstrated that ClC-5 is present in the human liver, brain and skeletal muscle.

On the contrary not all the 5′UTR isoforms are expressed in the various tissues. mRNA variants type 3, 2, 7 and alternative variant 4 appear to be the most abundant in the human kidney. 5′UTR exons that are commonly present among expressed isoforms are candidates for mutation analysis of Dent disease patients without genetic variation in the *CLCN5* coding region. Polymorphisms or rare variants might also reside in these regions that acting as modifier alleles and might explain the phenotypic heterogeneity of Dent disease not only in Dent disease 1 but also in Dent disease 2. We have demonstrated, in fact, that variants in both *OCRL* and *CLCN5* genes may act in concert in determining Dent disease phenotype variability [28].

Despite widespread expression of ClC-5, the Dent disease 1 phenotype is largely renal. Different 5′UTR ends present in various tissues may serve to differently regulate gene expression in response to physiological and pathological stimuli through mechanisms involving not only transcription but also translation efficiency. It is known, in fact, that the 5′UTR region has several roles in translational efficiency and translation inhibition probably through the interaction with the ribosome and specific DNA binding proteins or through some elements contained in non coding regions. So, it is possible that *CLCN5* mRNA levels do not correspond to ClC-5 protein level and actual ClC-5 functions.

Also of note is the presence of ClC-5 in the human brain and skeletal muscle. Although CNS and muscle impairment is common in Lowe syndrome, it has not been described in Dent disease 1 [11]. We recently evaluated a patient carrying a *CLCN5* mutation whose clinical symptoms suggested a Dent 2 phenotype or a mild Lowe phenotype (unpublished). Our findings point to the possibility that certain Dent cases with *CLCN5* disease-causing mutations might manifest extrarenal symptoms or a mild Lowe phenotype.

The tissues that are most similar, both in terms of abundance and expression pattern of *CLCN5* UTR isoforms are kidney, colon and testis. It is known that in rats and pigs ClC-5 is expressed in intestinal tissues that have endocytotic machinery [29,30]. As in renal proximal tubular and intercalated collecting duct cells, intestinal and colon epithelial cell ClC-5 is predominantly if not exclusively intracellular, located in densely packed endocytotic vesicles in rats [29]. Some authors have evaluated the role of ClC-5 in intestinal calcium absorption by directly regulating the expression of calcium transport proteins, such as TRPV 6 [30-33]. Although in humans the intestinal calcium absorption takes place mainly in small intestine, our data, albeit indirectly, can support the hypothesis that in Dent disease hypercalciuria may be due to increased intestinal absorption of calcium rather than decreased tubular re-absorption.

No phenotype associated with testicular dysfunction has been described so far in Dent disease patients. Future studies might be warranted to explore the possible role of ClC-5 in male infertility and to determine testicular function in Dent disease 1 patients, analogous to the role of the *CFTR* gene in male infertility [34].

## Conclusions

Our results confirm the structural complexity of *CLCN5* 5′UTR region. The presence of many different *CLCN5* 5′UTR ends as well as the selective use of alternative promoters can affect if/when and how the transcript is translated, by binding to different transcription factors and regulating translation efficiency. The meaning of this complexity, characterized by the presence of several *CLCN5* isoforms differentially-regulated in a tissue specific manner, likely in relation to physiological and/or pathological conditions, remains to be clarified. This complexity might explain aspects of the Dent disease phenotype and pathogenesis, and might obscure disease causing mutations or polymorphisms that influence disease expression. It will be interesting to analyze renal *CLCN5* isoforms and their expression levels in both normal and pathological conditions to identify and understand their physiologic and pathophysiologic roles.

## Competing interests

The Authors declare that they have no competing interests.

## Authors’ contributions

ET carried out the molecular genetic studies, the bioinformatic analysis and the interpretation of data and drafted the manuscript. AC has been involved in the cloning of PCR products obtained from RNA ligase-mediated rapid amplification of cDNA 5*’* ends PCR. LS critically evaluated the data. AF participated in interpretation of bioinformatic analysis data. JCL participated in revising the manuscript. FA participated in the design of the study and in drafting and revising the manuscript. All authors read and approved the final manuscript.

## Pre-publication history

The pre-publication history for this paper can be accessed here:

http://www.biomedcentral.com/1755-8794/7/41/prepub

## Supplementary Material

Additional file 1 **Primers used for amplification of *CLCN5* 5′ cDNA ends.** The table report the sequence of adaptor GeneRacer 5*’* primers supplied with the kit and the sequence of specific *CLCN5* gene antisense primers. Click here for file

Additional file 2 **Primers used for RT/PCR analysis of *CLCN5* mRNAs.** The mRNA species amplified, the amplicon length and the PCR conditions are reported. Some primer pairs co-amplified different *CLCN5* mRNA species. Click here for file

Additional file 3 **Primers used for Real-Time PCR analysis of 5′ *CLCN5* isoforms.** The mRNA species and the size of the PCR products are reported. Click here for file

Additional file 4 **Sequence of extended 5′ UTR ends of the known *CLCN5* transcripts (type 3, 4 and alternative 4) and sequence of the newly identified *CLCN5* 5′UTR variants (types 6–11).** Click here for file

Additional file 5 **RT/PCR analysis of the *CLCN5* 5′UTR isoforms in different human tissues.** Click here for file
