# Supplementary material for: Complexity of the 5′UTR region of the CLCN5 gene: eleven 5′UTR ends are differentially expressed in the human kidney
Source: BMC Med Genomics. 2014 Jul 7;7:41. doi: 10.1186/1755-8794-7-41 (PMC4105828; doi:10.1186/1755-8794-7-41)
Supplement: Additional file 1 — Primers used for amplification of CLCN5 5′ cDNA ends. The table report the sequence of adaptor GeneRacer 5’ primers supplied with the kit and the sequence of specific CLCN5 gene antisense primers. [file 1755-8794-7-41-S1.pdf]

ADDITIONAL FILE 1

Primers used for amplification of *CLCN5* 5' cDNA ends. The table report the sequence of adaptor GeneRacer 5' primers supplied with the kit and the sequence of specific *CLCN5* gene antisense primers.

PRIMER	*SEQUENCE* (5' → 3')
GeneRacer 5' primer	CGACTGGAGCACGAGGACACTGA
r RACE Ex 2	ACACCAGGGATTGGCTCCTCCAAGAA
GeneRacer 5' nested primer	GGACACTGACATGGACTGAAGGAGTA
r RACE Ex 2 bis	CTCTCGGTGCCTATCCCGGTCTCGAG
r RACE cn 1-2	ACCATTGTACGACTTGTCCTCTGTATTG
r RACE sz 1-2	CCATTGTACGACTTGTCCTCTGGTAGG
r RACE 1c	AGGGACAAGGCTTCCTCCAGACTGTTT
