# Supplementary material for: Complexity of the 5′UTR region of the CLCN5 gene: eleven 5′UTR ends are differentially expressed in the human kidney
Source: BMC Med Genomics. 2014 Jul 7;7:41. doi: 10.1186/1755-8794-7-41 (PMC4105828; doi:10.1186/1755-8794-7-41)
Supplement: Additional file 2 — Primers used for RT/PCR analysis of CLCN5 mRNAs. The mRNA species amplified, the amplicon length and the PCR conditions are reported. Some primer pairs co-amplified different CLCN5 mRNA species. [file 1755-8794-7-41-S2.pdf]

ADDITIONAL FILE 2

Primers used for RT/PCR analysis of CLCN5 mRNAs. The mRNA species amplified, the amplicon length and the PCR conditions are reported. Some primer pairs co-amplified different CLCN5 mRNA species.

EXON	***PRIMER PAIRS*** (5' → 3')	***mRNA variants***	***AMPLICON (bp)***	***T annealing (°C)***	***$MgCl_2$ (mM)***	***cyle numbers***
Ex I/II F<br>Ex IV/2 R	*f*: CCTCGGCGACAGAGCAAATC<br>*r*: TTGTCCTCTCCTACTTCTCGG	1 + 2	386 + 645	64	3	38
Ex 1a F<br>Ex 4 R	*f*: AGGCAGAGAATGCAGCAAGT<br>*r*: CATGCTCAGAGTTCCAGCAA	3	512	60	1.5	35
Ex 1b F<br>Ex 4 R	*f*: GACCCTTTTGTCTCCCTTCC<br>*r*: CATGCTCAGAGTTCCAGCAA	4 + alternative 4	586 + 720	60	1.5	35
5'UTR b F<br>Ex 4 R	*f*: GGCATGCTGACTTAGGTATTCA<br>*r*: CATGCTCAGAGTTCCAGCAA	6 + 7 +<br>4 + alternative 4	417 + 602 +<br>1560 + 1694	62	2	40
Ex I/II F<br>Ex VI/2 R	*f*: CCTCGGCGACAGAGCAAATC<br>*r*: CGACTTGTCCTCTGGAGTAGAA	8 + 9 + 10 + 11	584 + 715 + 843<br>+ 974	58	2	38
Ex 2-3 F<br>Ex 6R	*f*: TAGGCACCGAGAGATTACCAAT<br>*r*: GATGTTCCCACAGCAGCAAGC	Translated region	580	60	1.5	35
GAPDH F<br>GAPDH R	*f*: TGAAGGTCGGAGTCAACGGATTTGGT<br>*r*: CATGTGGGCCATGAGGTCCACCAC	GAPDH	983	60	1.5	30
