# Supplementary material for: Complexity of the 5′UTR region of the CLCN5 gene: eleven 5′UTR ends are differentially expressed in the human kidney
Source: BMC Med Genomics. 2014 Jul 7;7:41. doi: 10.1186/1755-8794-7-41 (PMC4105828; doi:10.1186/1755-8794-7-41)
Supplement: Additional file 3 — Primers used for Real-Time PCR analysis of 5′ CLCN5 isoforms. The mRNA species and the size of the PCR products are reported. [file 1755-8794-7-41-S3.pdf]

ADDITIONAL FILE 3

Primers used for Real Time PCR analysis of 5' *CLCN5* isoforms. The mRNA species and the size of the PCR products are reported.

EXON	*PRIMER* (5' → 3')	*mRNA variants*	*AMPLICON (bp)*
Ex 1a/2 F<br>Ex 2 R	*f*: TGTGATTGAAGAGGACAAGTCG<br>*r*: AGGTCCCTACACCAGGGATT	3	98
Ex 1b/2 F<br>Ex 2R	*f*: TTCCTACCAGAGGACAAGTCG<br>*r*: AGGTCCCTACACCAGGGATT	4	97
Ex 1b1/2 F<br>Ex 2R	*f*: CAATACAGAGGACAAGTCG<br>*r*: AGGTCCCTACACCAGGGATT	alternative 4	92
Ex c.1/2 F<br>Ex 2 R	*f*: ACCAATTAGGACAAGTCG<br>*r*: AGGTCCCTACACCAGGGATT	6	91
Ex c/2 F<br>Ex 2 R	*f*: CTGCCAAGAGGACAAGTCG<br>*r*: AGGTCCCTACACCAGGGATT	7	92
Ex IIa/III F<br>Ex III R	*f*: TGCCCCGAGTTTGGGGCTTT<br>*r*: AGAGCCTCTCAGTTTTGTAGGG	2 + 8 + 10	90
Ex IIb/III F<br>Ex III R	*f*: AGGAGTTTGTTTGGGGCTTT<br>*r*: AGAGCCTCTCAGTTTTGTAGGG	1 + 9 + 11	90
Ex 3/4 F<br>Ex 4 R	*f*: TTTATCAGGTTCGTTAGCTGG<br>*r*: CTCTTCAAAGGTGACATGCTC	Translated region	99
GAPDH F<br>GAPDH R	*f*: GAAGGTGAAGGTCGGAGT<br>*r*: TGGCAACAATATCCACTTTACCA	GAPDH	92
