# Supplementary material for: Complexity of the 5′UTR region of the CLCN5 gene: eleven 5′UTR ends are differentially expressed in the human kidney
Source: BMC Med Genomics. 2014 Jul 7;7:41. doi: 10.1186/1755-8794-7-41 (PMC4105828; doi:10.1186/1755-8794-7-41)
Supplement: Additional file 4 — Sequence of extended 5′ UTR ends of the known CLCN5 transcripts (type 3, 4 and alternative 4) and sequence of the newly identified CLCN5 5′UTR variants (types 6–11). [file 1755-8794-7-41-S4.pdf]

**ADDITIONAL FILE 4**

**Sequence of extended 5’ UTR ends of the known ClC-5 transcripts (type 3, 4 and alternative 4) and sequence of the newly identified *CLCN5* 5’UTR variants (types 6-11).** The alternative splice sites are indicated (*).

### mRNA variant 3

| | |
|---|---|
| | GCTGGTCCAAAGGTCTGGTCACGTGACTCCAACAGTGACATCACATTCTGACCGCCAGGG |
| EXTENDED | AACACTGGTAGTTCTTTAGGTGGCGTTTGTTGCTGTGAAAGGCAGAGAATGCAGCAAGTG |
| EXON 1a (242 bp) | CCCTTCTAAAGCACTTCAGACTGGGGCTTCTTTTGTAAAAGCTCCCCAACCTGAATGACC |
| | AAATGCGGGCTGCCAAGGTTATTTCTTCCTTGAGGCAGAAAAAAAGAAAACTGTGATTGA |
| | AG |
| | AGGACAAGTCGTACAATGGTGGAGGAATAGGTTCTTCAAATAGGATCATGGACTTCTTGG |
| EXON 2 (152 bp) | AGGAGCCAATCCCTGGTGTAGGGACCTATGATGATTTCAATACAATTGATTGGGTGAGAG |
| | AGAAGTCTCGAGACCGGGATAGGCACCGAGAG |

### mRNA variant 4 and alternative variant 4

| | |
|---|---|
| | ATGATTTGGACTCTGCAGAGCTTTTTCTTTCAAATGTGCACCTTGCCTGTGAAAGGCATG |
| | CTGACTTAGGTATTCAGAAGACTTACAATTGGTAAACCAATTGTAAGTACAGCTTGGGGT |
| | GTTCCTTCAAACTTTCCCCATGACATTGCTAAAGTTGTCTGTTTGGAGTTTACAGTGAAG |
| | ATGGGGATGCAGATGGGAGGAGAGGGAGTCAGCTCAGGCCCACTTTGGCCTCCTCTGCAG |
| | AGGGATTCATTACCAAACAGTCTGGAGGAAGCCTTGTCCCTGCCAAGGTTGGTGCTGCAA |
| | ATGTGAGCTTTGGCTGGGTGAATTAAATGATGGCATTACTCTGCACTTTTCCCCAGTGTG |
| | CATTTGCATAGATTCCTCAGTACTGTTTTGTATTTTGTGTTTAATAGCAAAATCTTATTC |
| | CATCAGATGATGTTGCTGAAATATTTTGAATCAAGTTTAAGCCTTGTGTGAGTATAGCTT |
| | GTGAGTTTAAAACTTTCTCCCCCATTCCCACCGTTCATCAAACAAATTTTGGTTCAGCTG |
| | ATATATACTGATTCTTGGATATGTGCTAGACACTGTAGAGAAGGGAACGATGAATCAGAC |
| EXTENDED | ATCAGGCTACCAGCAAAGAGCTTGTAGTCTAGAAAGGAAGATAAAGCATGCACAAAAGTA |
| EXON 1b (1001 bp) | ATTGTAATAGAAGTACCCAAAGCAAAGTAGATAAGGGACTATGATGGGTGGGCAGGTGGA |
| | TATGTTTCTTGCAGTTGAAGGATCAGGGAGGGACATTTCATCACTGAATCATACACTGTT |
| | CTTTAAATCTCTGCTTGTAAATCTTAGACTGTGCTGACATACTTAGAGTTCCTACAAGCC |
| | TGTTACATCAGAGATCAAGTCTTATACCTATTGAAGGGAAATTTGTACATTCCAGCCAAC |
| | TTAACTCGTGAAGAGGCCCACATGCTTTATTTGGAGTCCCCAGCCAGGCAAAAACCACTT |
| | CATAGGATTGAATTATTGCCTTATAAATGTAAAATGGTTCT |
| | TGATGTGATATGGCTGCAAGTGCCTTTGACCCTTTTGTCTCCCTTCCATAAACTGAAATA |
| | TGATGTGATATGGCTGCAAGTGCCTTTGACCCTTTTGTCTCCCTTCCATAAACTGAAATA |
| EXON 1b (244 bp) | CCTAAGCTGCTCCAACCTCCTTTTTGTCTTTTGTTTCATAAATCCTTTCCCATTGCACAT |
| | CAACTCCTGTCTCTCTTTGTACTGTCACTCTCATCTGTTGCTTTCCATTCACACTGCCTT |
| | TAGCCACTCATCATTTTGTGCCTACACCACAGAAACCTCTGAATGTAATGGATGTTCCTA |
| | CCAG* |
| | GTGACTGTATTTCTTTATCAACCCCAACTGTAGTCATCTGATAGTTTAAGGGCCCGCCTT |
| INTRON 1-2 (134 bp) | TTGGAACCAAAAAGAATGTATTAAAAATGTTAAGAATTTCTGAAGTACTAATGTCTATTT |
| | CTTGTTCAATACAG |
| | AGGACAAGTCGTACAATGGTGGAGGAATAGGTTCTTCAAATAGGATCATGGACTTCTTGG |
| EXON 2 (152 bp) | AGGAGCCAATCCCTGGTGTAGGGACCTATGATGATTTCAATACAATTGATTGGGTGAGAG |
| | AGAAGTCTCGAGACCGGGATAGGCACCGAGAG |

### mRNA variants 6 and 7

| | |
|---|---|
| | ATGATTTGGACTCTGCAGAGCTTTTTCTTTCAAATGTGCACCTTGCCTGTGAAAGGCATG |
| | CTGACTTAGGTATTCAGAAGACTTACAATTGGTAAACCAATT*GTAAGTACAGCTTGGGGT |

| | |
|---|---|
| | GTTCCTTCAAACTTTCCCCATGACATTGCTAAAGTTGTCTGTTTGGAGTTTACAGTGAAG |
| EXON c (287 bp) | ATGGGGATGCAGATGGGAGGAGAGGGAGTCAGCTCAGGCCCACTTTGGCCTCCTCTGCAG |
| | AGGGATTCATTACCAAACAGTCTGGAGGAAGCCTTGTCCCTGCCAAG |
| | AGGACAAGTCGTACAATGGTGGAGGAATAGGTTCTTCAAATAGGATCATGGACTTCTTGG |
| EXON 2 (152 bp) | AGGAGCCAATCCCTGGTGTAGGGACCTATGATGATTTCAATACAATTGATTGGGTGAGAG |
| | AGAAGTCTCGAGACCGGGATAGGCACCGAGAG |

## mRNA variants 8 and 9

| | |
|---|---|
| | GATACTCGCGAGGCGCCCGTCACATGAGCCGCGCGCCCTGCTTCGCTCCCCCTCCTCCCC |
| | GGCTGGGCTGTGTGAATGAAGCTCTGCCGGCTACGTGGGGCGCTCACTCAACTTGGTGTC |
| EXON I (197 bp) | CTGGACGCCAGAGCCGACCGAGCGCGCTGCCCACCGGCGGCGGACACGGGCTCCGCCGCT |
| | CCGGACCTCGGCGACAG |
| | AGCAAATCAGTTGCCTGGAGTTCCCAGTGAAGTTGTACCTGTGCCCGTGCGATAACGTCA |
| | GTGGAAGCTGCCCCGA*GTAAGTGAGGGAAATCTAGAAGTTTGGAGCATAAAAAACCAGC |
| EXON II (335 bp) | ACGAATCAACTTTACATAAACTTCATACATTTATTAGCAACTGCTCGGGTTTAACTTCAGG |
| | GATGTTAAATTTCGCCTAGTCCCACGGGACGATGATCATTACTTCTTAACTGCACGCGAA |
| | ATAGGAGAAGAAAAAAGGCAAATCAAAACAGACTGTCCCAGGTCTCAGAGAAGTCATCGG |
| | AGCGCTTTTTCCTGCTGAATTCACCCTAGGAGTTT |
| | GTTTGGGGCTTTAGCCCCTTGGAGAAAACAGGGCCACATAGCTGCCTTTCTATCAAGTCT |
| EXON III (144 bp) | CCCTACAAAACTGAGAGGCTCTGGGAAACTCAGCCTGTGACCCCAGCGTGGGTGAAGATA |
| | GGATCAAGATGGCCATGTGGCAGG |
| | GTGCCATGGATAACAGAGGCTTTCAGCAGGGGAGTTTTAGTAGCTTCCAGAACAGCTCCA |
| EXON IV (147 bp) | GTGATGAAGACCTGATGGACATTCCAGCAACCGCTATGGATTTCTCCATGAGAGATGATG |
| | TTCCTCCCTTAGACCGAGAAGTAGGAG |
| | AGCCAGGAGGGATTCTTAGATCTTCACTGTGAGAACTTGGTGGGGTTCCTGGATGTAGAG |
| EXON VI (194 bp) | CTTTGGTGTGGGAGCTCCCCTAAGACCGTGGTCCCCAAGAGTTTCTTGTACTCACGCCAG |
| | TCCACACTCAGCCTTCAGCAGCTTGTTCAAGTTGCCATTTGTGTTTCTACCAGCTCCAGC |
| | CATTTCTACTCCAG |
| | AGGACAAGTCGTACAATGGTGGAGGAATAGGTTCTTCAAATAGGATCATGGACTTCTTGG |
| EXON 2 (152 bp) | AGGAGCCAATCCCTGGTGTAGGGACCTATGATGATTTCAATACAATTGATTGGGTGAGAG |
| | AGAAGTCTCGAGACCGGGATAGGCACCGAGAG |

## mRNA variants 10 and 11

| | |
|---|---|
| | GATACTCGCGAGGCGCCCGTCACATGAGCCGCGCGCCCTGCTTCGCTCCCCCTCCTCCCC |
| | GGCTGGGCTGTGTGAATGAAGCTCTGCCGGCTACGTGGGGCGCTCACTCAACTTGGTGTC |
| EXON I (197 bp) | CTGGACGCCAGAGCCGACCGAGCGCGCTGCCCACCGGCGGCGGACACGGGCTCCGCCGCT |
| | CCGGACCTCGGCGACAG |
| | AGCAAATCAGTTGCCTGGAGTTCCCAGTGAAGTTGTACCTGTGCCCGTGCGATAACGTCA |
| | GTGGAAGCTGCCCCGA*GTAAGTGAGGGAAATCTAGAAGTTTGGAGCATAAAAAACCAGC |
| EXON II (335 bp) | ACGAATCAACTTTACATAAACTTCATACATTTATTAGCAACTGCTCGGGTTTAACTTCAGG |
| | GATGTTAAATTTCGCCTAGTCCCACGGGACGATGATCATTACTTCTTAACTGCACGCGAA |
| | ATAGGAGAAGAAAAAAGGCAAATCAAAACAGACTGTCCCAGGTCTCAGAGAAGTCATCGG |
| | AGCGCTTTTTCCTGCTGAATTCACCCTAGGAGTTT |
| | GTTTGGGGCTTTAGCCCCTTGGAGAAAACAGGGCCACATAGCTGCCTTTCTATCAAGTCT |
| EXON III (144 bp) | CCCTACAAAACTGAGAGGCTCTGGGAAACTCAGCCTGTGACCCCAGCGTGGGTGAAGATA |
| | GGATCAAGATGGCCATGTGGCAGG |
| | GTGCCATGGATAACAGAGGCTTTCAGCAGGGGAGTTTTAGTAGCTTCCAGAACAGCTCCA |
| EXON IV (147 bp) | GTGATGAAGACCTGATGGACATTCCAGCAACCGCTATGGATTTCTCCATGAGAGATGATG |
| | TTCCTCCCTTAGACCGAGAAGTAGGAG |
| | TGCCACCAAACTGTCTGCCAAAGTGCCAAAGTGGCTATACCATTTTGCATTCCTACCAGC |
| EXON V (131 bp) | TATGAATGAGAGTTCCTGTTAGTTTGCATCCTTGGTATTGTCAGTGTTTTGGATTTTAGC |
| | CATTCTAATAG |
| | AGCCAGGAGGGATTCTTAGATCTTCACTGTGAGAACTTGGTGGGGTTCCTGGATGTAGAG |
| EXON VI (194 bp) | CTTTGGTGTGGGAGCTCCCCTAAGACCGTGGTCCCCAAGAGTTTCTTGTACTCACGCCAG |
| | TCCACACTCAGCCTTCAGCAGCTTGTTCAAGTTGCCATTTGTGTTTCTACCAGCTCCAGC |
| | CATTTCTACTCCAG |
| | AGGACAAGTCGTACAATGGTGGAGGAATAGGTTCTTCAAATAGGATCATGGACTTCTTGG |
| EXON 2 (152 bp) | AGGAGCCAATCCCTGGTGTAGGGACCTATGATGATTTCAATACAATTGATTGGGTGAGAG |
| | AGAAGTCTCGAGACCGGGATAGGCACCGAGAG |
