# Supplementary material for: Complexity of the 5′UTR region of the CLCN5 gene: eleven 5′UTR ends are differentially expressed in the human kidney
Source: BMC Med Genomics. 2014 Jul 7;7:41. doi: 10.1186/1755-8794-7-41 (PMC4105828; doi:10.1186/1755-8794-7-41)

ADDITIONAL FILE 5

RT/PCR analysis of the *CLCN5* 5'UTR isoforms in different human tissues. A) common region of all isoforms (580 bp). **B)** mRNA variant 1 (645 bp) and mRNA variant 2 (386 bp). **C)** mRNA variant 3 (512 bp). **D)** mRNA variant 4 (586 bp) and mRNA alternative variant 4 (720 bp). **E)** mRNA variant 6 (417 bp) and variant 7 (602 bp). In colon is present a further PCR 782 bp long fragment which, once sequenced, was found to correspond to exon c 467 bp long, probably originated from a further alternative splicing of exon 1b1. The longest amplicon (1694 bp) represents the mRNA alternative variant 4. **F)** mRNA variant 8 (584 bp), 9 (715 bp), 10 (843 bp), and mRNA variant 11 (974 bp). **G)** Housekeping gene GAPDH (983 bp).

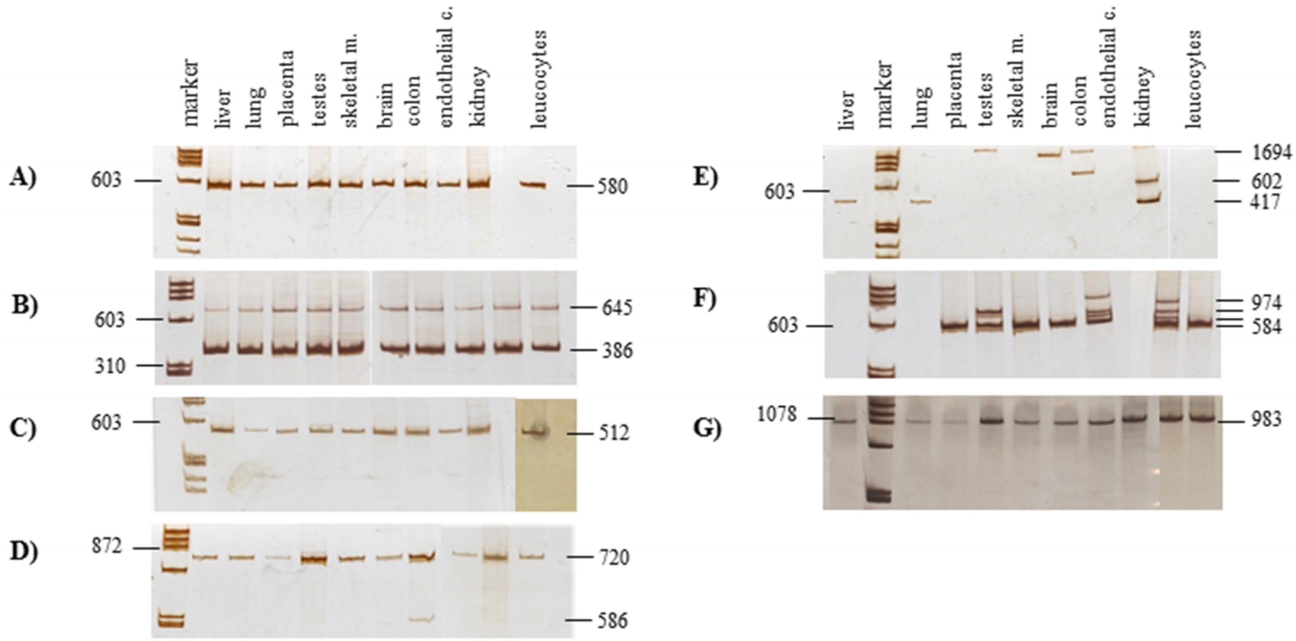

Supplement: Additional file 5 — RT/PCR analysis of the CLCN5 5′UTR isoforms in different human tissues. [file 1755-8794-7-41-S5.pdf]
